# Supplementary material for: Redox-enabled electronic interrogation and feedback control of hierarchical and networked biological systems
Source: Nat Commun. 2023 Dec 21;14:8514. doi: 10.1038/s41467-023-44223-w (PMC10739708; doi:10.1038/s41467-023-44223-w)
Supplement: Supplementary file 1 — Wang et al - Supplementary Information [file 41467_2023_44223_MOESM1_ESM.pdf]

Supplementary Information

Redox-enabled Electronic Interrogation and Feedback Control of Hierarchical and Networked Biological Systems

Authors:

Sally Wang[1,2,3*], Chen-Yu Chen[1,2,3*], John R. Rzasa[2], Chen-Yu Tsao[2,3], Jinyang Li[2,3], Eric VanArsdale[1,2,3], Eunkyoung Kim[2,3], Fauziah Rahma Zakaria[1,2,3], Gregory F. Payne[2,3], William E. Bentley[1,2,3†]

1 Fischell Department of Bioengineering, University of Maryland, College Park, Maryland, USA

2 Fischell Institute of Biomedical Devices, University of Maryland, College Park, Maryland, USA

3 Institute of Bioscience and Biotechnology Research (IBBR), University of Maryland, Rockville, Maryland, USA

Table of Contents:

Supplementary Figures 2

Supplementary Tables 25

References for Supplementary Information 27

Supplementary Figures

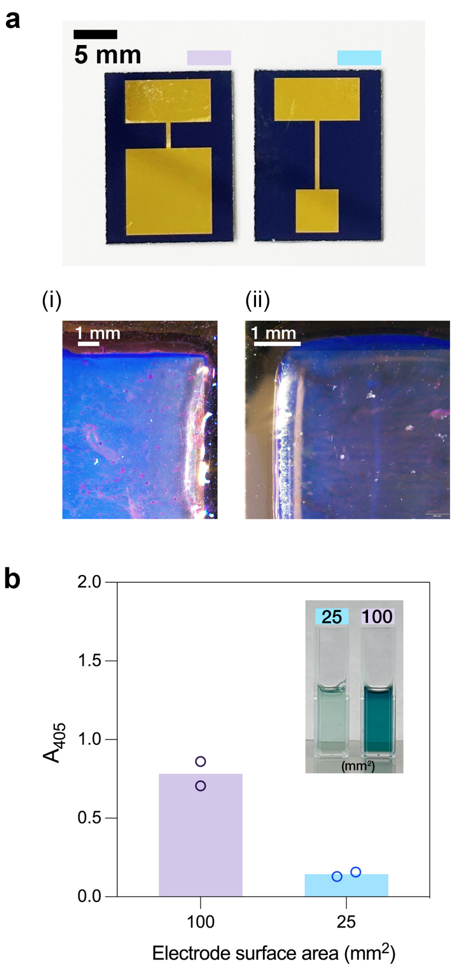

Supplementary Figure 1. Spatially electrodeposited HRP/gelatin hydrogel. (a) HRP/gelatin hydrogel electro-assembled on patterned gold electrodes with different surface area. (i) Brightview microscopy images, highlighting the edges, of HRP/gelatin hydrogel stained with Coomassie Blue on a square 10 × 10 mm gold electrode. (ii) Brightview microscopy images of HRP/gelatin hydrogel stained with Coomassie Blue on a square 5 × 5 mm gold electrode. **(b)** Activity of electrode-bound HRP as determined via ABTS assay. Absorbance reading at 405 nm after 4 min of incubation with ABTS+H_2O_2 assay solution in room temperature. Data are presented as mean (*n* = 2). Individual replicates are plotted as open circles.

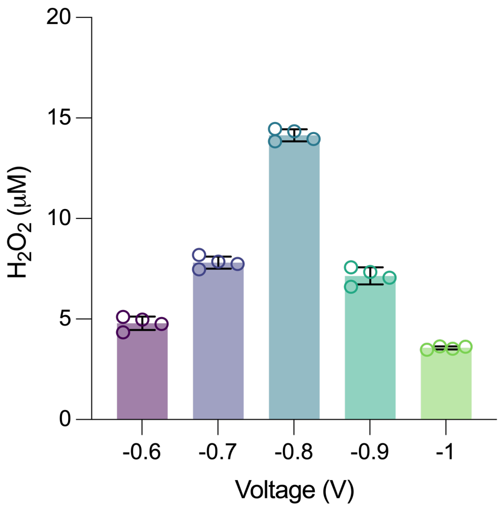

Supplementary Figure 2. H_2O_2 production under different applied voltage. Voltage was applied for 5 minutes to 150 μL of 20% LB. Data are presented as mean±s.d. (n = 4).

Notes – Addendums for Figure 2 in Main Manuscript

1. In **Fig. 2f**, we examined the difference in current obtained during electroinduction (-0.8 V) with or without exogenous oxygen supplement. Surface-assembled *E. coli* (NB101 + pOxy-sfGFP, OD_{600} = 6), submerged in 20% LB, was acclimatized in the custom environmental chamber at 34°C for 1.5 hours prior to electroinduction. The blue curve shows the output current without exogenous oxygen supplement (~0.5 μA, indicating minimal H_2O_2 generation), while the purple curve shows the current during which we supplied 0.4 ft^3/h (~0.01 m^3/h) of oxygen (starting from 0 s) through built-in tubing in the connector of the 4-well optoelectrochemical device. In the case of supplemented oxygen, it took about 2 minutes, but the measured current was observed to increase steadily. This profile is likely due to the active metabolism of the *E. coli* cells entrapped in the artificial biofilm that depleted the dissolved oxygen in the culture media. After two minutes, increased current shows that oxygen was present at the electrode surface, suggesting sufficient oxygen for respiratory function. Considering that the current for the controls without cells (both gel and no-gel) was always higher than the gel with cells, this indicates that the cells did indeed metabolize provided oxygen. Thus, we designed the 3D-printed optoelectrochemical device to include built-in tubing for exogenous oxygen supply. Schematic illustrations of the entry ports are shown below (in **Supplementary Fig. 3**). We believe that both oxygen diffusion and the gentle stirring (convection) caused by the airflow facilitated oxygen transport, and together, sufficient oxygen could be delivered to the electrode surface allowing both cell respiration and electroinduction.

2. In **Fig. 2e,** we chose -0.8 V as the applied voltage to generate hydrogen peroxide (H_2O_2) in the ITO-based electrochemical platform. As shown above (in **Supplementary Fig. 2**), applying -0.8 V to 150 µL of 20% LB in our custom device produced the highest level of H_2O_2. Additionally, we observed a visible color change to the electrode (from transparent to yellowish-brown) after applying -1 V to the ITO electrodes. The observed color change is also reported in previous studies and will likely affect the resistivity or other properties of the electrode[1].

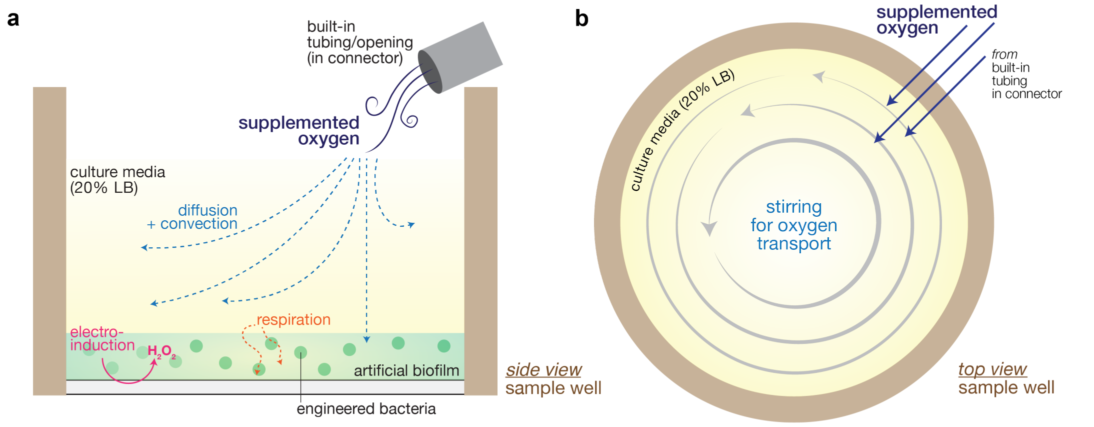

Supplementary Figure 3. Schematic of the mechanism for exogenous oxygen supply. (a) Side view of an individual sample well in the optoelectrochemical device. **(b)** Top view of an individual sample well in the optoelectrochemical device.

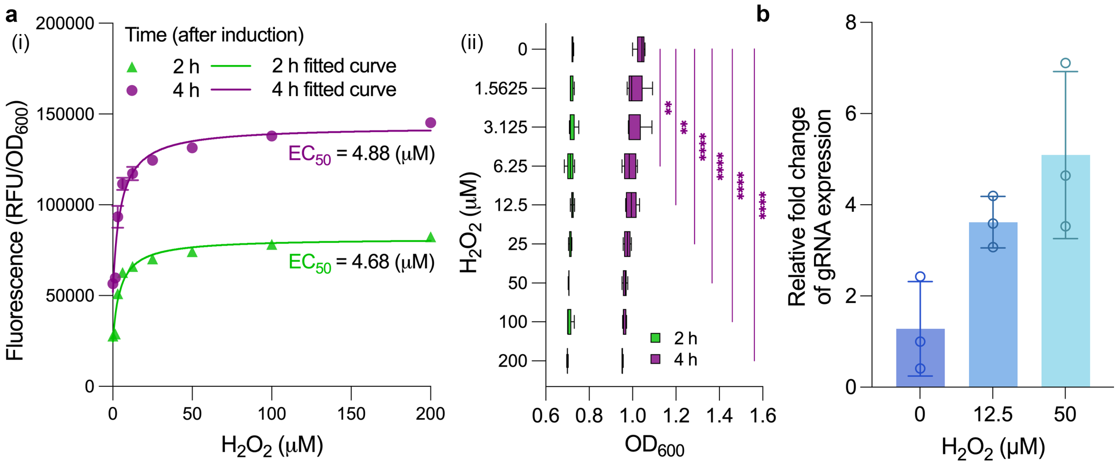

Supplementary Figure 4. H_2O_2-inducible CRISPR activation of *gfpmut2* (a) Fluorescence (i) and OD_{600} (ii) of NB101 harboring pSC-O108, pdCas9ω, and pMC-GFP measured at 2 hours (green) and 4 hours (purple) after H_2O_2 induction. Data are presented as mean±s.d. (n = 5). Filled triangle (2 h) and circles (4 h) represent individual replicates. Solid lines represent the fitted curves. EC_{50} values represent the half-maximal effective concentration of n = 5 H_2O_2 inductions of CRISPRa. Data in (ii) were analyzed by ordinary two-way ANOVA. *$P < 0.05$, **$P < 0.01$, ***$P < 0.001$, ****$P < 0.0001$. Exact P values can be found in the source data. **(b)** Relative fold change of gRNA (sg108) expression induced with various levels of H_2O_2. Data are presented as mean±s.d. (n = 3). Open circles represent individual replicates.

Notes

1. The fluorescence response (on a per cell level; we divided the total fluorescence from each homogenous culture by its OD_{600}) of eCRISPRa GFP cells (NB101 harboring pSC-O108, pdCas9ω, and pMC-GFP) induced with various concentrations of peroxide as indicated in **Supplementary Fig. 4a (i)**. We observed an increase in fluorescence intensity with peroxide and saw the response plateaued at higher peroxide concentrations (50-200 μM).
2. OD_{600} values of the samples noted above are also provided in **Supplementary Fig. 4a (ii)**. Although we found significant statistical differences in OD_{600} between the uninduced (0 μM) and induced (6.25-200 μM) cultures at hour 4, these differences are small (e.g., median OD_{600} of the uninduced and 200 μM sample are 1.04 and 0.95, respectively) and concluded peroxide (from 0-200 μM) exhibited minimal cytotoxicity to the eCRISPRa cells under current experimental conditions.

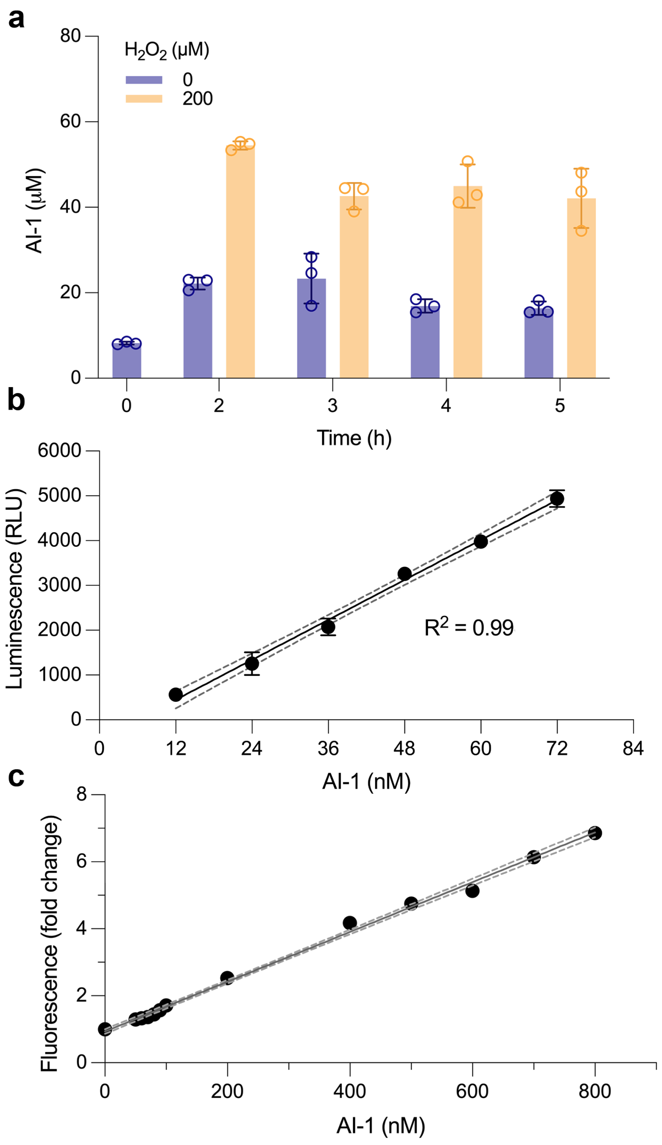

Supplementary Figure 5. H_2O_2-inducible CRISPR activation of *lasI* (a) AI-1 produced by NB101 harboring pSC-O108, pdCas9ω, and pMC-lasI-LAA after being induced by 0 µM (blue) and 200 µM (yellow) of H_2O_2. Data are presented as mean±s.d. (n = 3). Open circles represent individual replicates. **(b)** AI-1 bioassay standard curve. Data are presented as mean±s.d. (n = 3). Calibration curve was generated via linear interpolation of experimental data, and the dashed lines represent the 95% confidence interval. **(c)** Normalized fluorescence response from AI-1 inducible strain (NEB10β + LasR_S129T-GFPmut3). Data are presented as mean±s.d. (n = 3). The fitted curve was generated via linear interpolation of experimental data, and the dashed lines represent the 95% confidence interval.

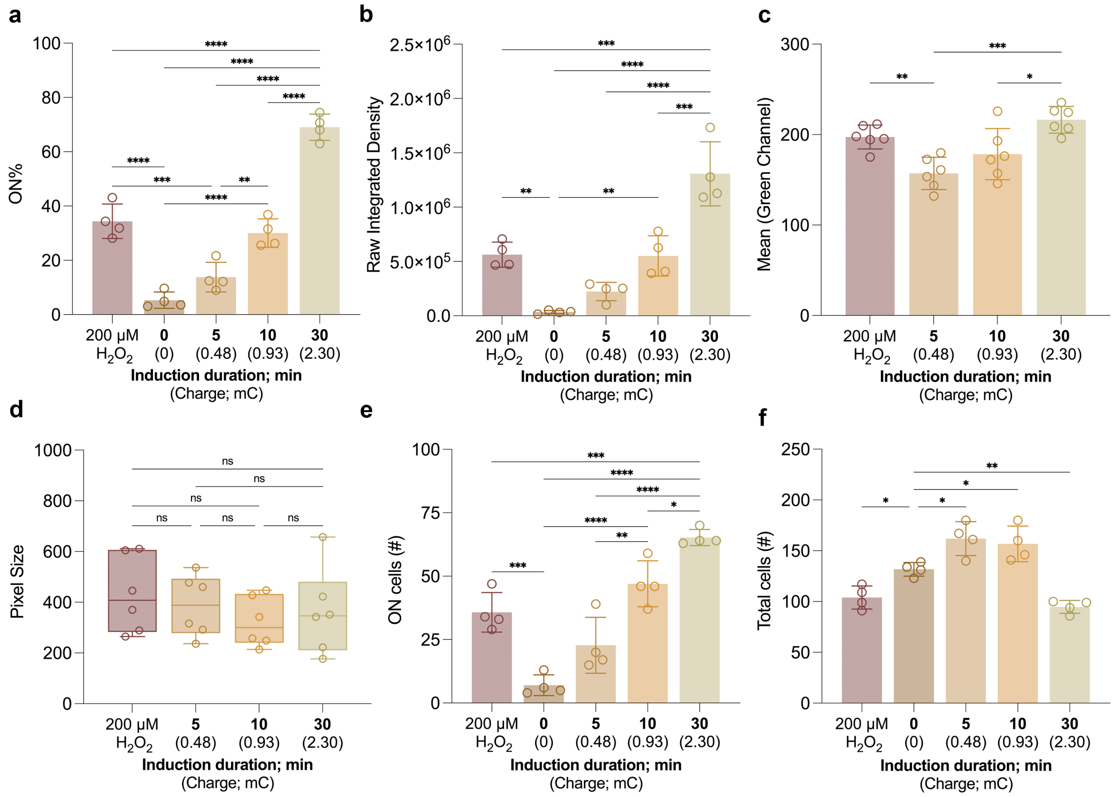

Supplementary Figure 6. Analysis of eCRISPRa confocal images depicted in Fig. 3c of the main manuscript. (a) Percentage of *E. coli* in the PEG-SH film that were activated through eCRISPRa. Data are adapted from **Fig. 3d**. **(b)** Raw integrated density obtained from FITC-channel images in **Fig. 3c**. **(c)** Mean gray value of individual "ON" cells ($n = 6$). **(d)** Pixel size of each "ON" cell assessed in **(c)**. Data are presented as box plots (center line at the median, upper bound at 75th percentile, lower bound at 25th percentile) with whiskers at minimum and maximum values. Each open circle represents one assessed "ON" cell. **(e)** Number of "ON" cells. **(f)** Number of total cells. Comparisons used ordinary one-way ANOVA. Comparisons between all samples are shown except for **(f)**, where only the comparisons between the uninduced (0) and the induced were included. Data are presented as mean±s.d. ($n = 4$; $n = 6$ for (c) and (d)). Individual replicates were indicated as open circles. $^{*}P < 0.05$, $^{**}P < 0.01$, $^{***}P < 0.001$, $^{****}P < 0.0001$, ns: not significant. Exact P values can be found in the source data.

Notes – Addendums for Figure 3 in Main Manuscript

1. In addition to **Fig. 3d**, we included the raw integrated density (sum of the values from each pixel) obtained from the FITC-channel confocal images in **Fig. 3c** as a representative of the total fluorescence recorded in each image (**Supplementary Fig. 6b**). We observed similar trends to the increase in ON% with increasing charge that is shown in **Fig. 3d**.
2. The green fluorescence intensity of a single "cell" (or, perhaps more accurately, single "cell-cluster") is shown in **Supplementary Fig. 6c** by analyzing the mean gray value from

six randomly-chosen cells in **Fig. 3**. The area (pixel size) of the cells is depicted alongside in **Supplementary Fig. 6d**. We found the size of “ON” cells is statistically uniform, and their fluorescence intensity exhibited a similar trend to the data depicting raw integrated density (**Supplementary Fig. 6b**).

3. The number of “ON” cells and total cells are shown in **Supplementary Fig. 6e** and **6f.** We observed similar trends between **6a** (ON%; “ON”/total cells), **6b** (total fluorescence in field of view), and **6e** (number of “ON” cells). Compared to the uninduced sample, we found the total cell count of the samples receiving 30-minute induction and 200 μM peroxide induction were slightly but significantly lower than the uninduced, suggesting again that peroxide exhibited low cytotoxicity. This observation also agrees with the OD_{600} data in **Supplementary Fig. 4a (ii).**
4. In **Fig. 3d-f**, we reported the total applied charge for each individual experiment. We believe the charge variations from the experiments results from slight differences in electrode surface area and material, potential deviation from the reference electrodes, and contents of the culture media.

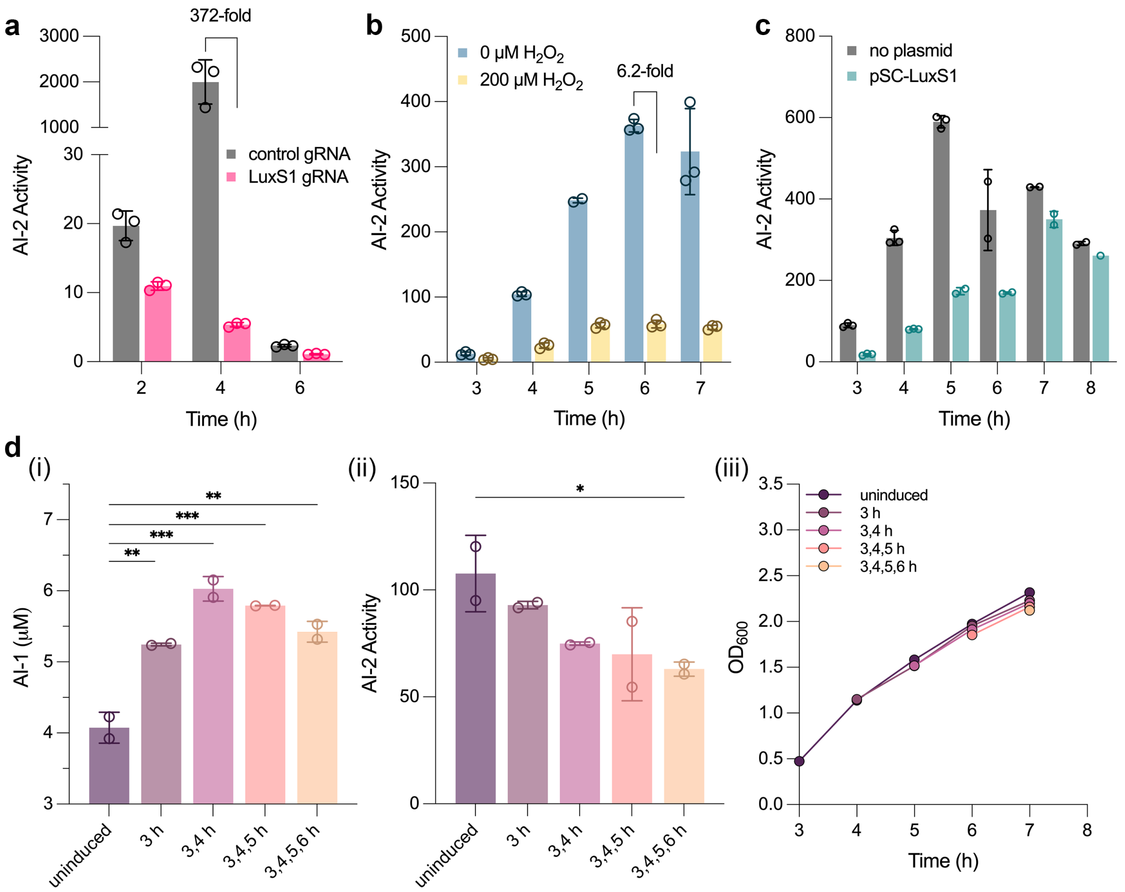

Supplementary Figure 7. H_2O_2-inducible *luxS* CRISPRi and multiplexed control of QS signaling. (a) Extracellular AI-2 activity of NB101 cells constitutively expressing either a control gRNA (grey) or *luxS*-specific gRNA LuxS1 (pink) at various time points after reinoculation. No glucose was added to LB media for inhibiting AI-2 uptake. **(b)** Extracellular AI-2 activity of NB101 cells carrying plasmids pSC-LuxS1 and pdCas9ω induced with 0 (blue) or 200 μM (yellow) of peroxide at OD_{600} = 0.4. Conditioned media samples were collected at various time points after re-inoculation. A final concentration of 0.8% (*w/v*) glucose was added to LB media for inhibiting AI-2 uptake. **(c)** Extracellular AI-2 activity profile of NB101 cells harboring no plasmid (grey) or eCRISPRi components (pSC-LuxS1+pdCas9ω; green) when assembled as "artificial biofilms". No glucose was added to 20% LB. No peroxide or charge is applied to the samples. **(d)** Measured (i) AI-1 concentration or (ii) AI-2 activity secreted from 'bilingual' cells 7 h post-reinoculation in liquid culture. Labels on the x-axis indicates when the cells received 200 μM H_2O_2 for inducing expression of both gRNAs. The growth curve (OD_{600}) of all experimental samples is shown in (iii). A final concentration of 0.8% (*w/v*) glucose was added to LB media for inhibiting AI-2 uptake. Filled circle represents the mean, and error bars represent the standard deviation (n = 2). Labels on the x-axis indicates when the cells received 200 μM H_2O_2 for inducing expression of both gRNAs. Data are presented as mean±s.d. (for (**a**) – (**c**) n = 3, for (**d**) n = 2). Open circle represents the individual replicates. In (d), comparisons

used one-way ANOVA, assuming the replicates were normally distributed, and P values were calculated between induced samples and the uninduced control. $*P < 0.05$, $**P < 0.01$, $***P < 0.001$, $****P < 0.0001$. Exact P values can be found in the source data.

Notes

1. Growth curves of the 'bilingual' cells are shown in **Supplementary Fig. 7d (iii)**. We observed attenuated final cell growth (at 7 h) as well as a relative decrease in growth rate for the cultures with more peroxide addition. Specifically, at hour 7, the uninduced samples (OD_{600} ~ 2.32) had a slightly higher OD_{600} than the samples with three (OD_{600} ~ 2.16) or four (OD_{600} ~ 2.12) inductions.

Notes – Addendums for Figure 4 in Main Manuscript

In **Fig. 4d**, we reported the total applied charge for the electroinduced samples. For the 0 h and 0&3 h samples, we applied -0.8 V for 30 minutes (resulting in a charge of 2.71 mC) to both cultures immediately after deposition. After 3 hours of incubation at 37°C, we administered an additional 30 minutes of -0.8 V electroinduction to the 0&3 h sample (resulting in an additional charge of 0.43 mC, making a total of 3.14 mC). We believe the diminished charge observed for the second application reflects the decrease of available oxygen at the electrode surface, because the cells were actively respiring and no oxygen was supplemented to the samples in this experiment.

Notes – Addendums for Figure 5 in the Main Manuscript

In **Fig. 5e**, we showed that the information "written" by WE1 (i.e., generated peroxide) can be "stored" in our electro-biochemical device and "retrieved" by WE2 at a later time. For this, we performed data writing (on WE1) and recording (on WE2) for a prolonged period (**Supplementary Fig. 8**). First, -0.8 V was applied to WE1 for 600 s to generate peroxide. 0 V was then applied to WE2 for peroxide detection, and we observed minimal decrease in WE2 current throughout the entire recording period (from 600 s to 3600 s for the #1 round). Next, this process (both data writing and recording) was repeated immediately (denoted the #2 round) and we saw an increase in WE2 current (throughout the entire 3600 s recording period), indicating that the increased peroxide level (from the #2 round of data writing) was stored and detected.

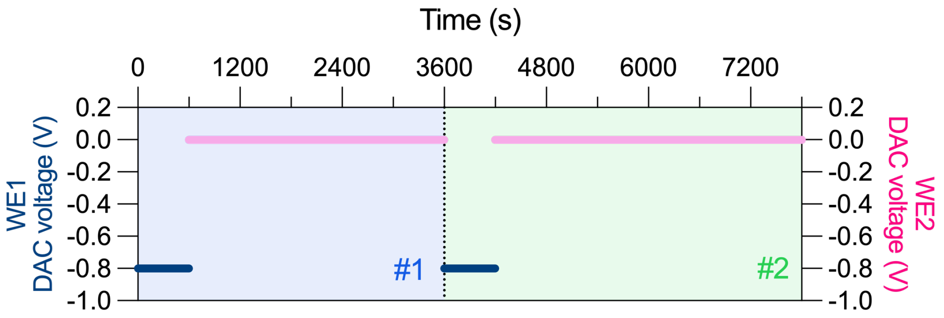

Supplementary Figure 8. Voltages applied on WE1 and WE2 of the electro-biochemical device (from **Fig. 5e**). Dark blue: applied voltage on WE1. Pink: applied voltage on WE2. Blue shaded area: round 1 (#1) of data writing and recording. Green shaded area: round 2 (#2) of data writing and recording.

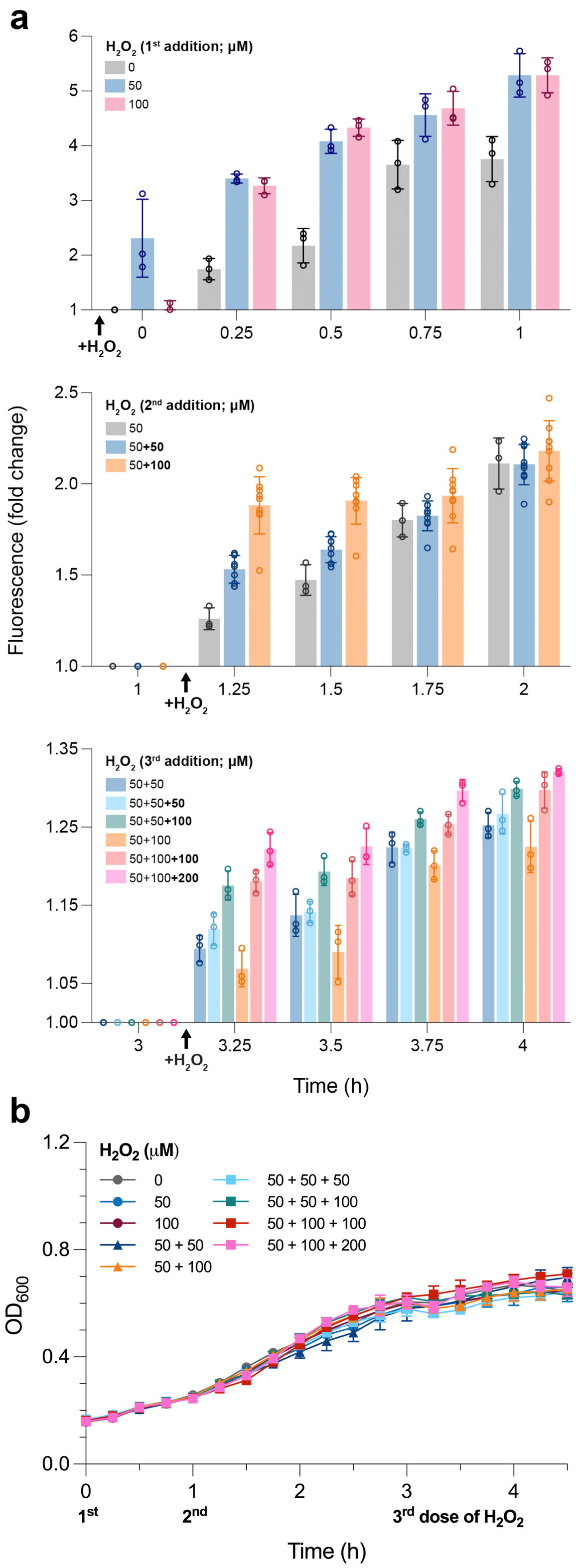

Supplementary Figure 9. Peroxide-induced gene expression is dynamic and can be controlled with repeated inductions. (a) Measured fluorescence from peroxide-reporter cells (NEB10β + pOxyRS-sfGFP-AAV). Cells were harvested at OD_{600} = 0.4 via centrifugation and re-inoculated to OD_{600} = 0.2 in 20% LB. Peroxide was added to each sample at indicated times

(arrows). Fluorescence values from 0 - 1 h was normalized to the fluorescence of the uninduced control at time 0. Fluorescence values from 1 - 4 h was normalized to each sample's fluorescence at the beginning of each cycle (i.e., 1 or 3 h). Data are presented as mean±s.d. ($n \geq 3$). Individual replicates were indicated by the open circles. **(b)** Growth curve of all experimental samples from repetitive inductions. Peroxide addition is indicated below its corresponding time. Filled circles (indicating one dose of peroxide), triangles (two doses), and squares (three doses) represent the mean. Error bars represent the standard deviation ($n = 3$).

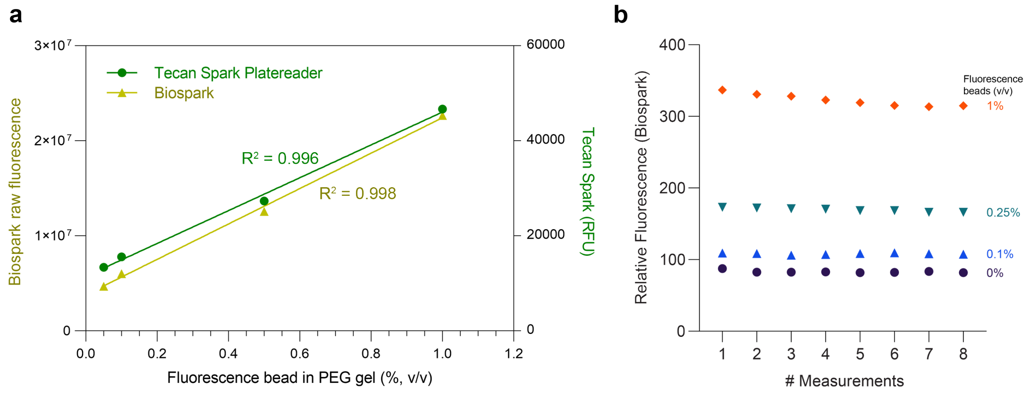

Supplementary Figure 10. Fluorescence measurements of the BioSpark system: deposited film. (a) Green fluorescent particles were co-deposited with PEG-SH to various concentrations for simulating fluorescence emitted from engineered bacteria in the 'artificial biofilm'. The resulted hydrogel was submerged in 150 µL of 20% LB, which is identical to the cell experiments. Fluorescence was read using BioSpark (light green) and the Tecan Spark® (dark green) microplate reader. Linear responses for both were observed. **(b)** Relative measured fluorescence from fluorescent particles/PEG-SH hydrogel. The four samples were read in the sequence of 0% → 0.1% → 0.25% → 1% consecutively for 8 cycles.

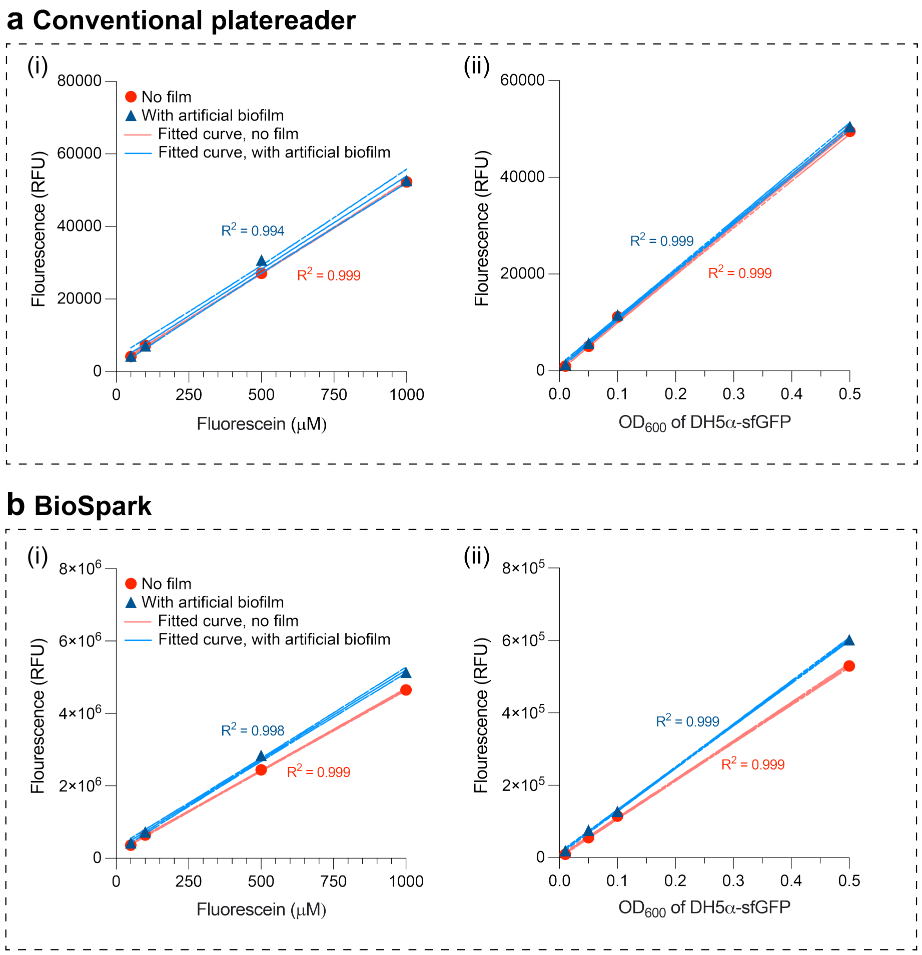

Supplementary Figure 11. Fluorescence measurements of the BioSpark system: supernatant. (a) Fluorescence measurements by the Tecan Spark® microplate reader. (i) 150 μL of fluorescein solution (in 10 mM NaOH) at various concentrations were added to the wells with (blue triangles) or without (red circles) a pre-deposited artificial biofilm for simulating fluorescence emitted from the fluids (no particles) above gel. (ii) 150 μL of DH5α-sfGFP cells (constitutively expressing sfGFP) diluted to various OD_{600} with 20% LB were added to the wells with (blue triangles) or without (red circles) a pre-deposited artificial biofilm for simulating fluorescence emitted from planktonic cells. **(b)** Fluorescence measurements by BioSpark. (i) 150 μL of fluorescein solution (in 10 mM NaOH) at various concentrations were added to the wells with (blue triangles) or without (red circles) a pre-deposited artificial biofilm for simulating fluorescence emitted from the fluids (no particles) above gel. (ii) 150 μL of DH5α-sfGFP cells (constitutively expressing sfGFP) diluted to various OD_{600} with 20% LB were added to the wells with (blue triangles) or without (red circles) a pre-deposited artificial biofilm for simulating fluorescence emitted from planktonic cells. The artificial biofilm was deposited with OD_{600} = 6 of the CRISPRa *lasI* cells that is identical to the cell experiments. Data are presented as mean±s.d. ($n = 3$). Calibration curve was generated via linear interpolation of experimental data, and the dashed lines represent the 95% confidence interval. Linear responses for all were observed.

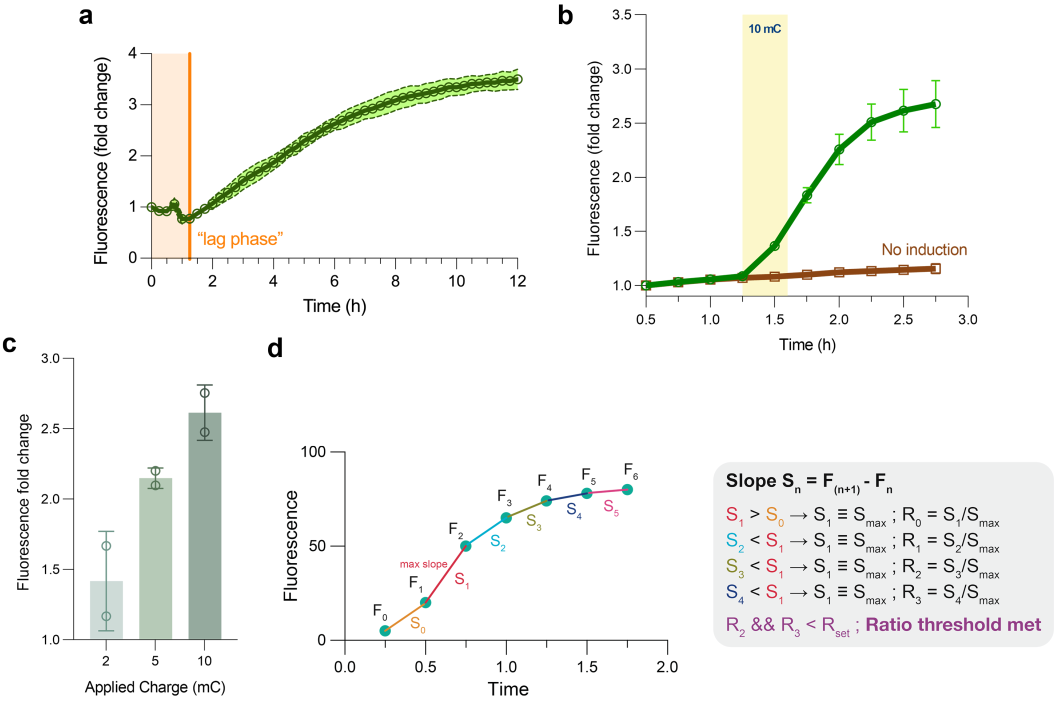

Supplementary Figure 12. Development of a custom algorithm to achieve automated electrical control of gene expression. (a) "Growth curve" of engineered *E. coli* entrapped in artificial biofilm from the measured fluorescence of DH5α-sfGFP cells co-deposited with PEG-SH. These cells constitutively express GFP in the absence of inducer; their expression is a surrogate for cell number. A "lag-phase" of approximately 1.25 hours was consistently observed during which there was no increase in fluorescence, suggesting that there was no growth during this period. Open circles represent the mean fluorescence, and the dashed curves represent the s.d. from individual replicates (n = 4). **(b)** Representative gene expression dynamic for in-film peroxide reporters (NEB10β + pOxy-sfGFP). The yellow zone (vertical band) indicates period during which voltage was applied for electroinduction. The uninduced negative control is also shown (in brown). Note that there are four wells in the device operated in parallel (hence two biological replicates for both experimental sample and negative control). Each well is interrogated with a moving optical fluorescence probe. Fluorescence from each well is measured three times and the average is reported. The elapsed time of the entire process (to measure fluorescence for all four wells) is typically less than two minutes. Open circles and squares (indicated) represent the mean, and error bars represent the s.d. of individual biological replicates (n = 2). **(c)** Fold change in fluorescence before and after electroinduction. Bar height represents the mean and error bars represent the s.d. (n = 2). Individual replicates are indicated by the open circles. **(d)** Basis data pairing for phenomenological algorithm underpinning gene expression assessment (see **Methods**). Since the fluorescence measurements were taken at fixed time intervals, we defined the slope as the difference between two neighboring

fluorescence measurements. The algorithm stores and updates the value of the maximum slope. It also computes the ratio between the current slope and the maximum slope (S_{max}). If two consecutive ratios fall below the user-set limit (expressed as a ratio), the algorithm considers the threshold met and initiates electro-induction via the potentiostat. The S_{max} will, in turn, return to 0 and a new cycle will begin.

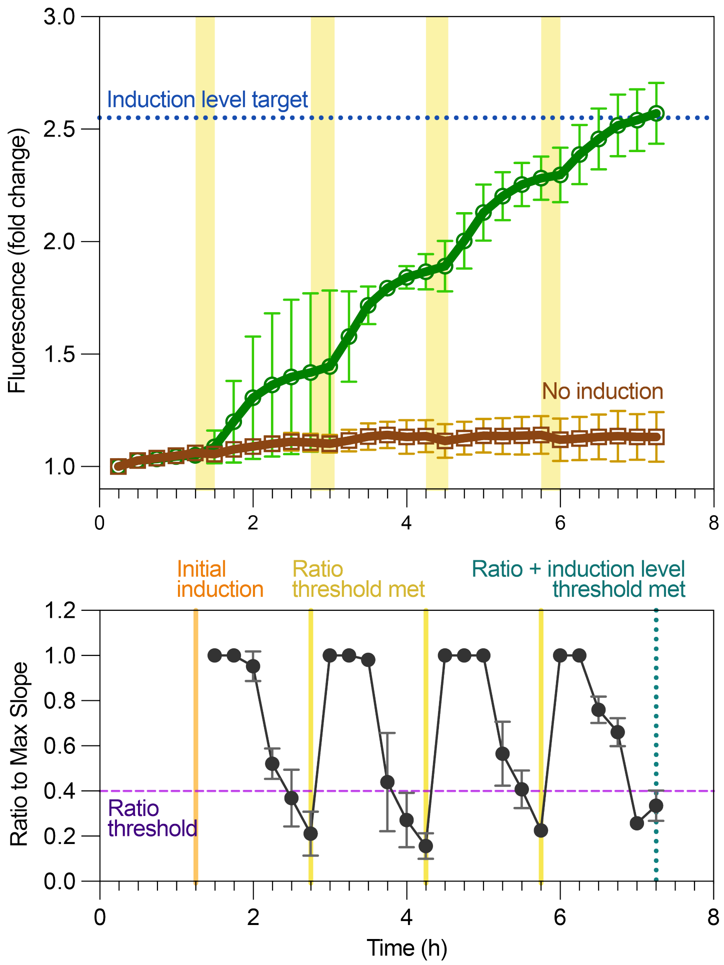

Supplementary Figure 13. Automated dynamic control of electro-induced gene expression. *Top*: Fluorescence level of the artificial biofilm containing peroxide reporters (NB101+pOxy-sfGFP). The experiment was automatically terminated when the fluorescence exceeded the induction level target (blue dotted line). The brown dataset indicates the fluorescence level of the negative control to which no induction voltage was applied. Yellow zones indicate the duration over which the induction voltage (-0.8 V) was applied. Note, to provide sufficient peroxide and metabolic activity, each experimental and negative control samples was supplemented with 0.4 ft^3/h of oxygen (flow rate determined over many runs). A total charge of 2 mC was applied for each induction (width of yellow zone indicates the amount of time to reach 2mC). Open circles and squares represent the mean of biological replicates (n = 2).
Bottom: Ratio of slope, S, to S_{max} computed by our custom algorithm. Ratio threshold (purple dashed line) was set at 0.4. The orange line indicates when the algorithm applied the initial induction voltage (1.25 h). Yellow lines indicate that two consecutive ratios were below the set point threshold, thus meeting the ratio threshold and triggering the potentiostat to apply induction voltage. The teal dotted line indicates when both the ratio threshold and the induction level threshold were met, hence no voltage was applied, and the experiment ended. For all figures, the error bars represent the s.d. of individual replicates (n = 2).

Notes – Addendums for Figure 6 of the Main Manuscript

In **Fig. 6c (i)**, the thickness of the yellow zones demonstrates the time over which electroinduction was administered (i.e., peroxide was generated). During this period, we also supplied 0.4 ft^3/h of oxygen to both the experimental samples and the negative controls. Since the total charge in each yellow zone was kept constant (2 mC), the increased time (from ~12 min to ~20 min) of voltage (-0.8 V) application to reach this charge also reflects the decreasing oxygen level at the electrode surface. As shown in **Supplementary Fig. 14**, the generated current within each electroinduction cycle is dynamic and increases along the electroinduction period due to the supplied oxygen. We note also that the total current gradually decreased as the cycles progressed. We believe this reflects a decreasing oxygen level at the electrode commensurate with increasing cell number (**Supplementary Fig. 12a**) present in the artificial biofilm. On the other hand, the transient increases in fluorescence from the uninduced negative control may be due to added in oxygen availability from the oxygen line since oxygen was only supplemented during electroinduction periods.

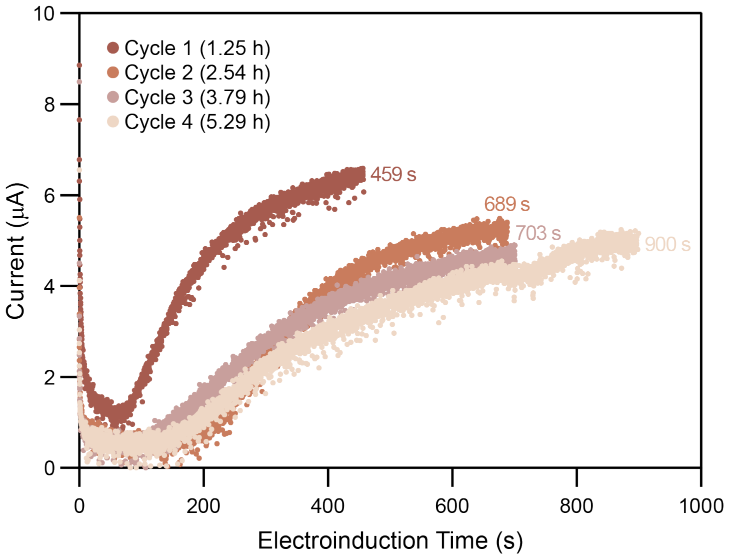

Supplementary Figure 14. Generated current during electroinduction (from **Fig. 6c** (i)). From dark to light brown: electroinduction cycles 1 to 4.

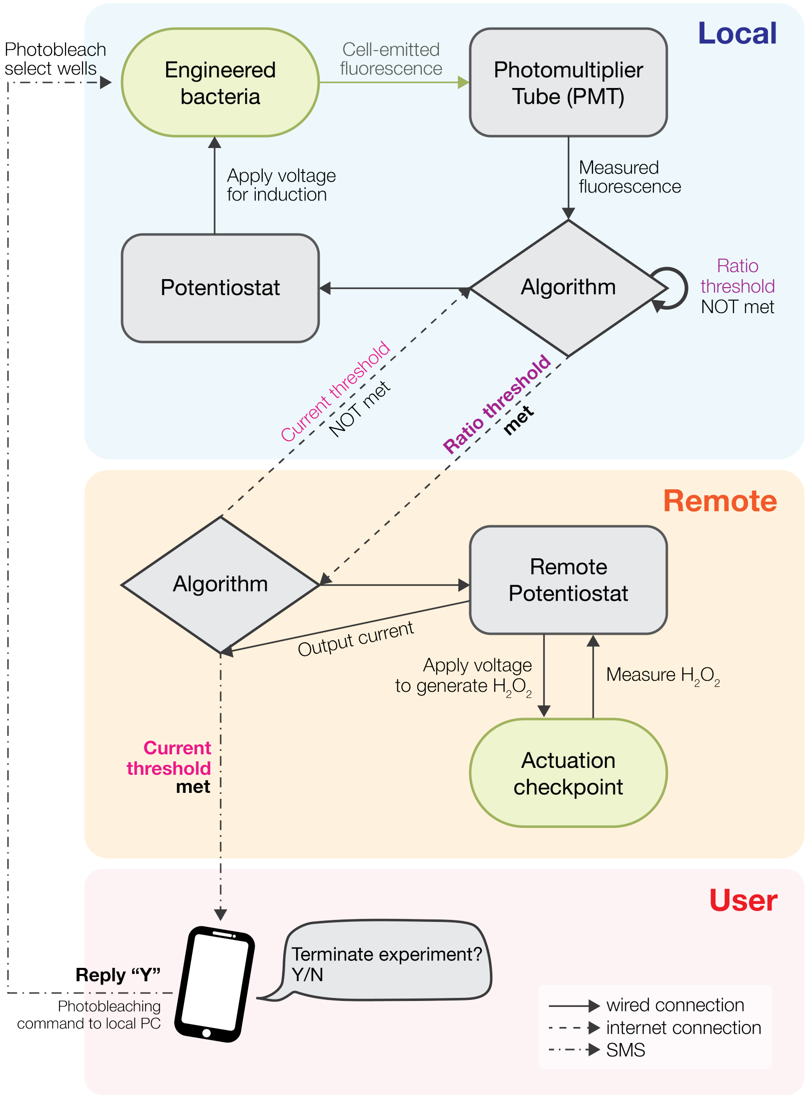

Supplementary Figure 15. System diagram of the integrated network representing the "Internet of Life". *Local:* Emitted cell fluorescence is measured three times (over 2 minutes) by the fluorescence module in the BioSpark system every 15 minutes and fed to the custom algorithm for fluorescence/expression level tracking. The local algorithm (see **Supplementary Fig. 12**) (i) stores and updates the S_{max}, (ii) computes the slope ratio, and (iii) compares the current ratio to the user-defined ratio threshold. When two consecutive slope ratios are less than or equal to the threshold value, the local algorithm considers the ratio threshold met and sends a message to the PC situated at the remote location wirelessly through the internet.
Remote: When the bio-electrochemical platform (connected to a PC) (denoted "actuation checkpoint") located at the remote location receives the message from the local BioSpark system, the remote algorithm then commands the remote potentiostat to run a pre-set program. -0.8 V will first be applied on WE1 for peroxide generation, followed by 0 V on WE2 for peroxide detection. The remote algorithm then compares the value of the output current to that of the user-defined current threshold: if the output current does not exceed the threshold, a message is returned (via internet) to the local BioSpark system to administer electroinduction

immediately after the upcoming fluorescence measurement (for a user-defined duration or charge). The elapsed time for this occurrence was typically 15 minutes. Otherwise, the remote algorithm sends a SMS verification message to alert the human users and seek permission to terminate the experiment.

User: Human users can respond to a query by sending text messages on a mobile phone (by replying “Y “or “N”) to indicate whether the experiment should be terminated or not. If replied with “Y”, BioSpark initiates the photobleaching program as a demonstration to destroy the expression product.

Notes – Addendums for Figure 7 of the Main Manuscript

In **Fig. 7a (i)**, like **Fig. 6c (i)**, the thickness of the yellow zones demonstrates the time over which electroinduction was administered (i.e., peroxide was generated). These datasets are from two independent experiments but are very similar in the result. Like in **Fig. 6c**, during this period, 0.4 ft^3/h of oxygen was supplied to both the experimental samples and the negative controls. We observed similar electroinduction and expression behavior here: (i) the increase in electroinduction duration along the time suggests the decrease in oxygen level at the electrode surface; and (ii) the transient increases in fluorescence from the negative control might also be due to changes in oxygen availability since oxygen was only supplemented during electroinduction. The generated current during electroinduction is shown in **Supplementary Fig. 16**. Here again, the observed duration of the 2 mC charge increased in time as the culture progressed to the higher fluorescence levels and cell densities.

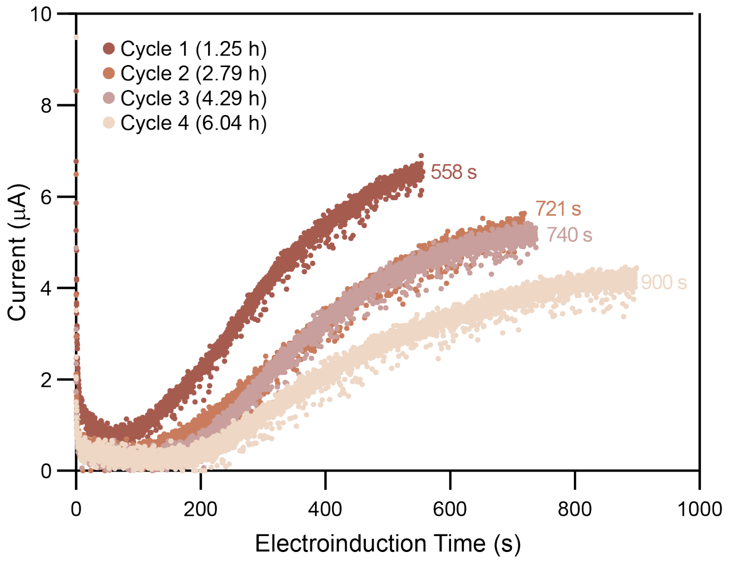

Supplementary Figure 16. Generated current during electroinduction (from **Fig. 7a** (i)). From dark to light brown: electroinduction cycles 1 to 4.

Notes – Addendums for Figure 7 of the Main Manuscript (continued)

To terminate the experiment, we used the fluorescence measurement module in BioSpark to photobleach the GFP. Schematic illustrations of the two different functions of the fluorescence measurement module are shown in **Supplementary Fig. 17**. For fluorescence measurements, a pulsed (0.07 ms) excitation light (at 469 ± 17.5 nm) was directed to the samples for 100 times and concurrently the optical probe also collected the emission light (at 525 ± 19.5 nm). For photobleaching, a continuous excitation light (469 ± 17.5 nm) was directed to the samples (for 15 min) instead.

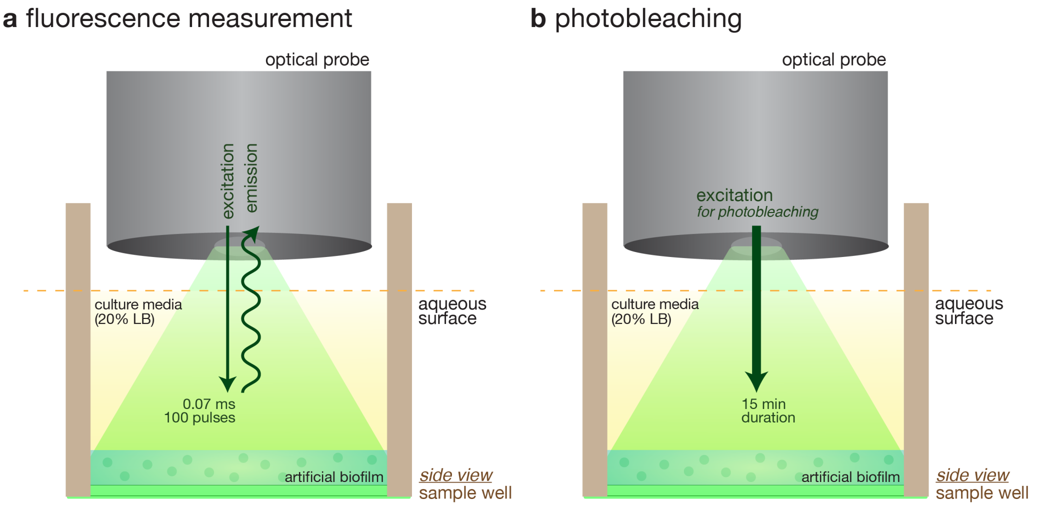

Supplementary Figure 17. Schematic of the fluorescence measurement module in BioSpark. (a) Fluorescence measurements **(b)** photobleaching

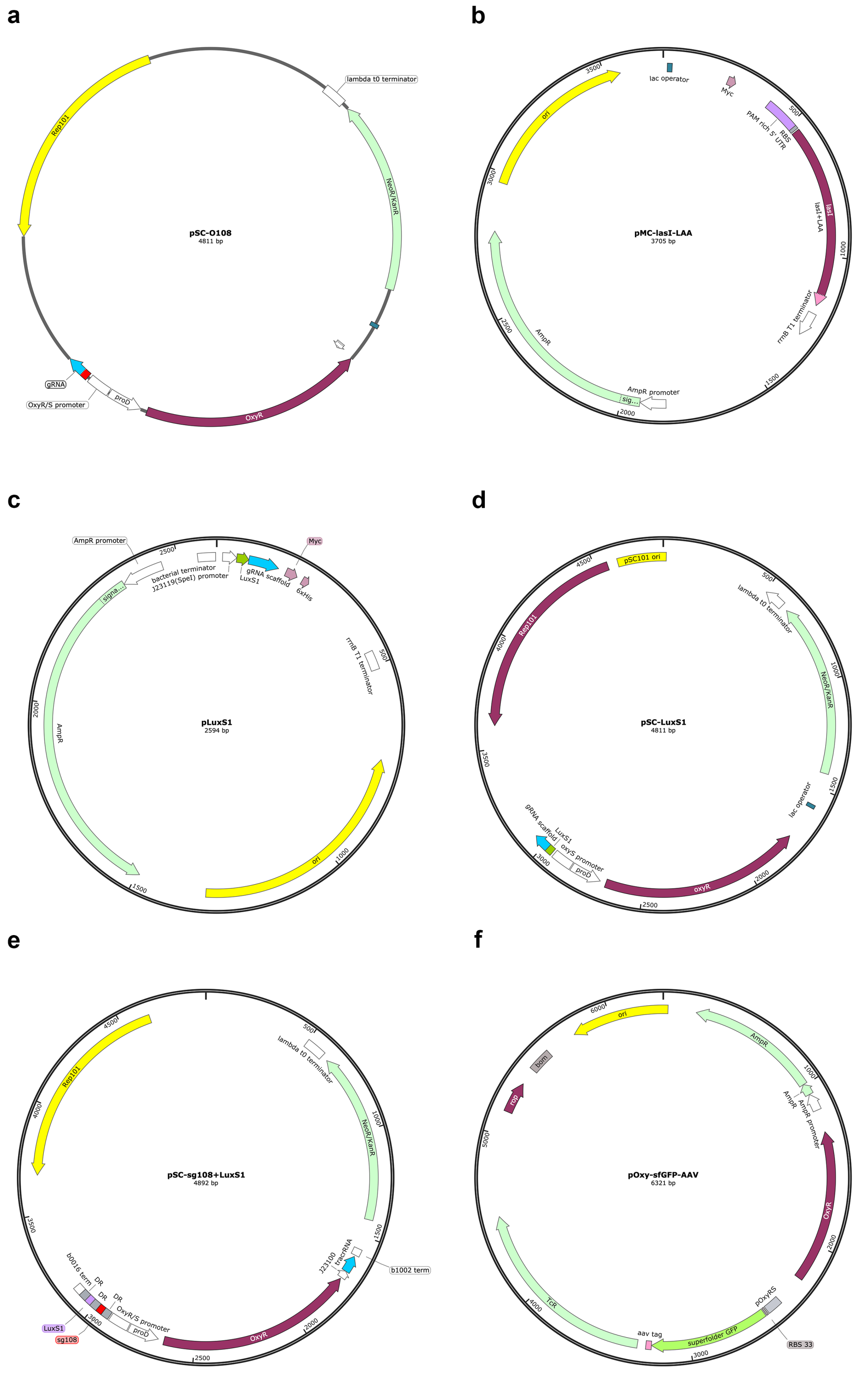

Supplementary Figure 18. Plasmids maps. (a) pSC-O108 **(b)** pMC-lasI-LAA **(c)** pLuxS1 **(d)** pSC-LuxS1 **(e)** pSC-sg108+LuxS1 **(f)** pOxy-sfGFP-AAV

Supplementary Tables

Supplementary Table 1. Strains and plasmids used in this study.

**Strains**		
**Name**	**Genotype**	**Reference**
NEB10β	*E. coli Δ(ara-leu) 7697 araD139 fhuA ΔlacX74 galK16 galE15 e14- ϕ80dlacZΔM15 recA1 relA1 endA1 nupG rpsL* (Str^R) *rph spoT1 Δ(mrr-hsdRMS-mcrBC)*	New England Biolabs
DH5α-sfGFP	*E. coli* DH5α *attTn7::mTn7ΦsfGFP*	This study
NB101	*E. coli* ZK126 Δ*rpoZ*	Bhokisham *et al.*[2]
JLD271	*E. coli* K-12 *ΔlacX74 sdiA271::Cam*	Lindsay *et al.*[3]
BB170	*V. harveyi luxN*::Tn5	Surrette *et al.*[4]
**Plasmids**		
**Name**	**Description**	**Reference**
pSC-S108gRNA	pSC101 ori, Kan^r, *soxR*, *soxRSp*, spacer 108, gRNA scaffold	Bhokisham *et al.*[2]
pOxy-LacZ-laa	pBR322 ori, Amp^r, proD pomoter, RBS 31, *oxyR*, *oxyRS* promoter, RBS 30, *lacZ* fused with LAA ssRA tag	Terrell *et al.*[5]
pSC-O108	pSC101 ori, Kan^r, proD promoter, RBS31, *oxyR*, *oxySp*, spacer 108, gRNA scaffold	This study
pdCas9ω	ω was inserted into C termini of dCas9 in pdCas9-bacteria (Addgene plasmid # 44249), p15A ori, pLtetO-1, Cm^r	Bhokisham *et al.*[2]
pMC-GFP	pWJ89 with pBR322 and Amp^r instead of pSC101 ori and Kan^r	Bhokisham *et al.*[2]
pMC-lasI-LAA	pMC-GFP *lasI* with the LAA ssRA tag instead of *gfpmut2*	This study
pLasR_S129T-GFPmut3	pSB1A2 backbone, pTetR-LasR(S129T)-pLuxR-GFP	This study
pS1gRNA	*soxS* specific gRNA spacers S1 inserted into pgRNAbacteria (Addgene plasmid # 44251) with pBR322 ori, Amp^r, BBa_J23119 promoter	Bhokisham *et al.*[2]
pControlgRNA	Control spacer (from Bikard *et al.*[6]) in pgRNA-bacteria (Addgene plasmid # 44251) with pBR322 origin, Amp^r, J23119 promoter	Bhokisham *et al.*[2]
pLuxS1	pS1gRNA with *luxS* specific gRNA spacer LuxS1 instead of S1	This study
pSC-LuxS1	pSC-O108 with *luxS* specific gRNA spacer LuxS1 instead of 108	This study
pSC-sg108+LuxS1	pSC101 ori, Kan^r, BBa_J23100 promoter, tracrRNA, b1002 terminator, proD promoter, RBS 31, *oxyR*, *oxyRS* promoter, DR, spacer 108, DR, spacer LuxS1, DR, b1006 terminator	This study
pOxy-sfGFP	pBR322 ori, Amp^r, proD pomoter, RBS 31, *oxyR*, *oxyRS* promoter, RBS 33, *sfGFP*	Li, Wang *et al.*[7]
pOxy-sfGFP-AAV	AAV ssRA tag inserted to *sfGFP* in pOxy-sfGFP	This study

Supplementary Table 2. Primers used in this study.

**Name**	**Sequence (5'-3')**
SW01	TGTTTGACAGCTTATCATC
SW02	GCGTCCGGCGTAGAGTCGTGTGAGCAATTATCAG
SW03	CACACGAGCGTGTTGTGG
SW04	GTAGAGAAAAAAGCACCGACTCGG
SW05	GTCGGTGCTTTTTTCTCTACCTCTACGCCGGACGCATC
SW06	TTCCACAACACGCTCGTGTGTCGTGTGAGCAATTATCAGTCAG
SW07	GAAGCTATCTAACAAGTTTTAGAGCTAGAAATAGCAAGTTAAAATAAG
SW08	ACAGTCGATCATACCTCGTGTGTCGTGTGAGCA
SW09	CTAACGGATCCGCTTTTTTCTCTACCTCTACGCC
SW10	CTAAGCTCGAGTGTGAGCAATTATCAGTCAGAATG
SW11	GAAGCTATCTAACAAGTTTTAGAGCTAGAAATAGC
SW12	ACAGTCGATCATACCACTAGTATTATACCTAGGAC
SW13	GGATCCCATGGTACGCGTG
SW14	CTAGATTTCTCCTCTTTAAAGGAATTCGC
SW15	TTTAAAGAGGAGAAATCTAGATGATCGTACAAATTGGTCGG
SW16	GCACGCGTACCATGGGATCCTTATTATCACGCTGCAAGGG
SW17	TGATGCGGTAGTTTATCAC
SW18	AACAGCAGCAGCGTAGTTTTCGTCGTTTGCTGCTTTGTAGAGCTCATCCAT
SW19	GCGTGTTGTGGAAGATCCG
SW20	CGACTCGGTGCCACTTTTTC

Supplemental Information References:

1 Ma, Z., Li, Z., Liu, K., Ye, C. & Sorger, V. J. Indium-Tin-Oxide for High-performance Electro-optic Modulation. *Nanophotonics* **4**, 198-213, doi:doi:10.1515/nanoph-2015-0006 (2015).

2 Bhokisham, N. *et al.* A redox-based electrogenetic CRISPR system to connect with and control biological information networks. *Nat Commun* **11**, 2427, doi:10.1038/s41467-020-16249-x (2020).

3 Lindsay, A. & Ahmer, B. M. Effect of sdiA on biosensors of N-acylhomoserine lactones. *J Bacteriol* **187**, 5054-5058, doi:10.1128/JB.187.14.5054-5058.2005 (2005).

4 Surette, M. G., Miller, M. B. & Bassler, B. L. Quorum sensing in *Escherichia coli, Salmonella typhimurium*, and *Vibrio harveyi*: a new family of genes responsible for autoinducer production. *Proc Natl Acad Sci U S A* **96**, 1639-1644, doi:10.1073/pnas.96.4.1639 (1999).

5 Terrell, J. L. *et al.* Bioelectronic control of a microbial community using surface-assembled electrogenetic cells to route signals. *Nat Nanotechnol* **16**, 688-697, doi:10.1038/s41565-021-00878-4 (2021).

6 Bikard, D. *et al.* Programmable repression and activation of bacterial gene expression using an engineered CRISPR-Cas system. *Nucleic Acids Res* **41**, 7429-7437, doi:10.1093/nar/gkt520 (2013).

7 Li, J. *et al.* Interactive Materials for Bidirectional Redox-Based Communication. *Advanced Materials* **33**, 2007758, doi:https://doi.org/10.1002/adma.202007758 (2021).
