# Supplementary material for: Redox-enabled electronic interrogation and feedback control of hierarchical and networked biological systems
Source: Nat Commun. 2023 Dec 21;14:8514. doi: 10.1038/s41467-023-44223-w (PMC10739708; doi:10.1038/s41467-023-44223-w)
Supplement: Supplementary file 3 — Description of Additional Supplementary Files [file 41467_2023_44223_MOESM3_ESM.pdf]

Description of Additional Supplementary Files

Supplementary Data 1: Sequences of relevant genetic parts
