# Supplementary material for: Redox-enabled electronic interrogation and feedback control of hierarchical and networked biological systems
Source: Nat Commun. 2023 Dec 21;14:8514. doi: 10.1038/s41467-023-44223-w (PMC10739708; doi:10.1038/s41467-023-44223-w)
Supplement: Supplementary file 5 — Reporting Summary [file 41467_2023_44223_MOESM5_ESM.pdf]

nature portfolio

Corresponding author(s): Dr. William E. Bentley

Last updated by author(s): Nov 27, 2023

Reporting Summary

Nature Portfolio wishes to improve the reproducibility of the work that we publish. This form provides structure for consistency and transparency in reporting. For further information on Nature Portfolio policies, see our Editorial Policies and the Editorial Policy Checklist.

Statistics

For all statistical analyses, confirm that the following items are present in the figure legend, table legend, main text, or Methods section.

n/a	Confirmed	
☐	☒	The exact sample size (n) for each experimental group/condition, given as a discrete number and unit of measurement
☐	☒	A statement on whether measurements were taken from distinct samples or whether the same sample was measured repeatedly
☐	☒	The statistical test(s) used AND whether they are one- or two-sided<br>*Only common tests should be described solely by name; describe more complex techniques in the Methods section.*
☒	☐	A description of all covariates tested
☒	☐	A description of any assumptions or corrections, such as tests of normality and adjustment for multiple comparisons
☐	☒	A full description of the statistical parameters including central tendency (e.g. means) or other basic estimates (e.g. regression coefficient) AND variation (e.g. standard deviation) or associated estimates of uncertainty (e.g. confidence intervals)
☐	☒	For null hypothesis testing, the test statistic (e.g. F, t, r) with confidence intervals, effect sizes, degrees of freedom and P value noted<br>*Give P values as exact values whenever suitable.*
☒	☐	For Bayesian analysis, information on the choice of priors and Markov chain Monte Carlo settings
☒	☐	For hierarchical and complex designs, identification of the appropriate level for tests and full reporting of outcomes
☒	☐	Estimates of effect sizes (e.g. Cohen's d, Pearson's r), indicating how they were calculated

Our web collection on statistics for biologists contains articles on many of the points above.

Software and code

Policy information about availability of computer code

Data collection	Tecan Spark software was used to collect fluorescence and luminescence data from the Tecan Spark microplate reader. Olympus cellSens Standard was used to obtain fluorescence microscopy images in Fig. 2b. Zeiss ZEN 3.9 was used to obtain confocal microscopy images in Fig. 2c, 3c. CH Instruments software was used to collect electrochemical data (e.g., current and charge) for Fig. 2, 3, and 4. Custom software was used to collect fluorescence and electrochemical data in Fig. 5-7.
Data analysis	All raw data were analyzed with Microsoft Excel. The processed data were then imported to GraphPad Prism 10 for generating graphs. ImageJ Version 1.54g was used to analyze images in Fig. 3c to generate Fig. 3d and Supplementary Fig. 6. All Statistical analyses were performed with GraphPad Prism 10. For Fig. 6 and 7, our custom algorithm processed the raw fluorescence and electrochemical data, perform calculations, and make decisions based on its analyzed results. Certain current data (in Fig. 2f and Supplementary Fig. 14,16) were re-sampled (from 10 ms to 100 ms) using our custom software. Current data (in Fig. 2f and Fig. 5e) was smoothed using our custom software. The complete packaged code (written in C#) for the custom software can be obtained upon request to the corresponding author.

For manuscripts utilizing custom algorithms or software that are central to the research but not yet described in published literature, software must be made available to editors and reviewers. We strongly encourage code deposition in a community repository (e.g. GitHub). See the Nature Portfolio guidelines for submitting code & software for further information.

Data

Policy information about availability of data

All manuscripts must include a data availability statement. This statement should provide the following information, where applicable:

- Accession codes, unique identifiers, or web links for publicly available datasets
- A description of any restrictions on data availability
- For clinical datasets or third party data, please ensure that the statement adheres to our policy

Sequences of relevant genetic parts from all plasmids generated in this study can be found in the Supplementary Information and Supplementary Data 1. All source data are provided with this paper. All other data and information are available upon request from the corresponding author.

Research involving human participants, their data, or biological material

Policy information about studies with human participants or human data. See also policy information about sex, gender (identity/presentation), and sexual orientation and race, ethnicity and racism.

Reporting on sex and gender	Human participants and human data were not involved/used in this study.
Reporting on race, ethnicity, or other socially relevant groupings	Human participants and human data were not involved/used in this study.
Population characteristics	Human participants and human data were not involved/used in this study.
Recruitment	Human participants and human data were not involved/used in this study.
Ethics oversight	Human participants and human data were not involved/used in this study.

Note that full information on the approval of the study protocol must also be provided in the manuscript.

Field-specific reporting

Please select the one below that is the best fit for your research. If you are not sure, read the appropriate sections before making your selection.

☒ Life sciences ☐ Behavioural & social sciences ☐ Ecological, evolutionary & environmental sciences

For a reference copy of the document with all sections, see nature.com/documents/nr-reporting-summary-flat.pdf

Life sciences study design

All studies must disclose on these points even when the disclosure is negative.

Sample size	No sample size calculations are performed for all experiments.<br>For Fig. 2b: two biological replicates (hydrogels) are casted and assayed for either HRP activity or QS activity. Colorimetric HRP activity assay was performed with one technical replicate. All QS bio-assays were performed with at least two biological replicates of the same sample.<br>For Fig. 2e: Four samples (representing individual samples) are collected in each well (each well underwent different durations of electroinduction) and assayed with the colorimetric peroxide assay kit. For each assessment, two technical replicates were performed. We reported the four replicates because the experiment does not involve biological samples.<br>For experiments performed in liquid culture (Supplementary Fig. 3,5,7,9): at least three sets of individual biological replicates were performed.<br>For QS experiments performed in our custom optoelectrochemical device (Fig. 3&4): due to the constraint in device size, at least two sets of individual biological replicates were collected to run QS (AI-1 or AI-2) bio-assays. All QS bio-assays were performed with at least two biological replicates of the same sample.<br>For Fig. 6&7: due to the constraint in the size of our custom optoelectrochemical device and BioSpark, we designated two wells in the optoelectrochemical device for experimental samples (two biological replicates) and two wells for controls. Fluorescence from each well was measured three times.
Data exclusions	No data were excluded from the analyses.
Replication	The reproducibility of this study and its results was demonstrated through the biological replicates presented in the figures and the supplementary information.
Randomization	All experimental cultures were reinoculated from the same overnight culture, and assigned randomly as individual experimental samples or controls.
Blinding	Investigators were not blinded during data collection and analyses. All fluorescence, QS activity, HRP activity, and electrical current data are reported as raw or relative/normalized values. For data generated from confocal microscopy, identical image settings were applied to all samples. We believe there is not much room for interpretation that could be subjective.

Reporting for specific materials, systems and methods

We require information from authors about some types of materials, experimental systems and methods used in many studies. Here, indicate whether each material, system or method listed is relevant to your study. If you are not sure if a list item applies to your research, read the appropriate section before selecting a response.

Materials & experimental systems

n/a	Involved in the study
☒	☐ Antibodies
☒	☐ Eukaryotic cell lines
☒	☐ Palaeontology and archaeology
☒	☐ Animals and other organisms
☒	☐ Clinical data
☒	☐ Dual use research of concern
☒	☐ Plants

Methods

n/a	Involved in the study
☒	☐ ChIP-seq
☒	☐ Flow cytometry
☒	☐ MRI-based neuroimaging
